# Antihypertensive drug-associated adverse events in osteoarthritis: a study of a large real-world sample based on the FAERS database

**DOI:** 10.3389/fphar.2024.1404427

**Published:** 2024-09-02

**Authors:** Zijian Guo, Jingkai Di, Zhibo Zhang, Shuai Chen, Xingjia Mao, Zehua Wang, Zehui Yan, Xiaoke Li, Zui Tian, Changjiang Mu, Changxin Xiang, Chuan Xiang

**Affiliations:** ^1^ Department of Orthopedic, The Second Hospital of Shanxi Medical University, Taiyuan, China; ^2^ Department of Basic Medicine Sciences, Department of Orthopaedics of Sir Run Run Shaw Hospital, Zhejiang University School of Medicine, Hangzhou, China; ^3^ College of Biomedical Engineering, Taiyuan University of Technology, Taiyuan, China

**Keywords:** hypertension, osteoarthritis, pharmacovigilance, valsartan, FAERS

## Abstract

**Background:**

Hypertension is a common complication in patients with osteoarthritis (OA). There is increasing interest in the relationship between hypertension and OA. However, hypertension has been reported to negatively affect symptoms and quality of life in patients with OA. Therefore, treating hypertension is crucial for patients with OA. However, there is a lack of real-world studies on the effects of medications for treating hypertension on OA.

**Methods:**

Data from the FAERS database from January 2004 to December 2023 were extracted for disproportionality analyses, and proportional reporting ratios (PRRs) were used to assess the association between medications for hypertension and all types of arthritis. Adverse event signals were identified and determined using reporting odds ratios (RORs) Adverse event signals were considered to have occurred if a drug-induced adverse event was recorded more than or equal to 3 and the lower limit of the ROR confidence interval was more than 1. We selected five classes of drugs including, calcium channel blockers (CCBs), angiotensin converting enzyme inhibitors (ACEIs), angiotensin receptor blockers (ARBs), thiazide diuretics and β-blockers and representative drugs were analysed for osteoarthritis-related adverse reactions, and age and gender subgroups were analysed for drugs of significance. We also analysed the occurrence of AEs in relation to time using the Weibull distribution.

**Results:**

In terms of overall data, we found significant OA adverse reaction signals only for ARBs among the five drug classes.ARB AEs for spinal osteoarthritis (ROR 4.64, 95% CI 3.62–5.94), osteoarthritis (ROR 3.24 95% CI 2.82–3.72) and gouty arthritis (ROR 3.27 95% CI 1.22–8.75) were the three adverse reactions with the loudest signals. Next, we found that valsartan had strong osteoarthritis adverse reaction signals among the three ARBs, namely, irbesartan, cloxartan, and valsartan. We also analysed age and gender subgroups and found that osteoarthritis signals were strongest in the 18–65 and 65+ population, while females seem to be more prone to valsartan-related OA AEs.

**Conclusion:**

ARBs, especially valsartan, have significant positive signals for OA AEs. Therefore, ARB drugs, especially valsartan, should be used with caution when treating patients with OA combined with hypertension.

## 1 Introduction

Osteoarthritis (OA) is a major cause of reduced quality of life for patients and is the sixth leading cause of disability globally. Various risk factors, including age, smoking, body mass index (BMI), low-density lipoprotein (LDL), and alcohol consumption, contribute to OA (Abramoff and Caldera, 2020). OA is most prevalent in the elderly, where it is the leading cause of chronic pain, functional impairment, and poor quality of life (Peat et al., 2001). It has been demonstrated that patients with OA are at a higher risk of developing hypertensive disorders (Dillon et al., 2006; Veronese et al., 2018). Additionally, patients with OA have a greater risk of experiencing cardiovascular events and all-cause mortality compared to non-OA patients (Nüesch et al., 2011; Haugen et al., 2015; Kendzerska et al., 2017). Previous studies have also revealed the association between hypertension and the development of knee OA (Zhang et al., 2017; Lo et al., 2022; Shi and Schlenk, 2022). Therefore, treating hypertension is crucial for improving the quality of life and prognosis of patients with OA.

Currently, there are five classes of first-line drugs used to treat hypertension: Calcium channel blockers (CCBs), angiotensin converting enzyme inhibitors (ACEIs), angiotensin receptor blockers (ARBs), thiazide diuretics and beta-blockers (Whelton et al., 2017; Al-Makki et al., 2022). It is important to use clear and concise language when discussing medical treatments, and to avoid complex terminology that may be difficult for the reader to understand. Several studies have investigated the potential association between certain drugs and the development of OA. Zhou et al. reported that ACEIs may have potential for the treatment of knee OA (Zhou et al., 2020). Li et al. (2021a) found that the use of calcium channel blockers was associated with narrowing of the knee joint space. Driban et al. (2016) observed symptomatic changes in patients with knee OA who were taking thiazides. It is important to note that these findings are not conclusive and further research is needed to fully understand the relationship between these drugs and OA. However, there are no studies analysing the effect of hypertensive medications on OA using large databases.

FDA Adverse Event Reporting System (FAERS) is a public database of adverse reaction reports from healthcare professionals, manufacturers, and consumers. It contains information on adverse reactions to drug use, including when they occur and their outcomes. The database is used by the FDA for record-keeping and post-market safety oversight of drugs (Sharma and Kumar, 2022; Javed and Kumar, 2024). This study collected testing records of ACEIs, ARBs, CCBs, beta-blockers, and hydrochlorothiazide medications to count the signal values of adverse reactions associated with OA. The aim was to provide a large-sample study of medications used in the treatment of hypertension and joint damage, and effective recommendations for the treatment of patients with OA combined with hypertension.

## 2 Materials and methods

### 2.1 Data sources

The study’s data was sourced from the open-source FAERS database, which adheres to the International Safety Reporting Guidelines (ICH E2B) published by the International Conference on Harmonisation (ICH). The FAERS database is updated quarterly and covers seven areas: patient demographics and management information (DEMO), drug information (DRUG), adverse event codes (REAC), patient outcomes (OUTC), reporting source (RPSR), treatment start and end dates (THER) associated with the reported drug, and indication for use (INDI) (Li H. et al., 2021). Additionally, we provided information on duplicate entries. Each adverse event (AE) was coded using the Medical Dictionary of Regulatory Activities (MedDRA, version 26.1) Preferred Terminology (PT). The PT is a unique descriptor for individual medical concepts, such as diagnoses and symptoms. The hierarchy also includes High-Level Terminology (HLT) and High-Level Group Terminology (HLGT). HLGTs are categorised into systemic organ categories according to etiology, site of disease or purpose. HLGTs are classified into systemic organ classes (SOCs) based on etiology, site of disease, or purpose (Brown et al., 1999). For this study, musculoskeletal and connective tissue diseases were selected for SOCs, and arthritis-related disease names were selected for PT. The search criteria used by MedDRA were applied.

### 2.2 Data process

This study collected report files from Q1 2004 to Q4 2023, excluding reports with documentation errors and missing data. Reports where the role cod attribute of the drug in the DRUG file was designated as ‘PS’ (major suspicion) were screened to improve the confidence of the adverse event (AE) analyses. Representative drugs from the five classes of hypertensive drugs were selected for analysis. The ACEIs we selected included captopril, enalapril and fenazopyridine. The CCB class is represented by nifedipine, diltiazem, and verapamil. Losartan, valsartan, and irbesartan are representative drugs of the ARB class (Burnier and Brunner, 2000). Beta-blockers are represented by propranolol, metoprolol, and labetalol. Thiazide diuretics are represented by hydrochlorothiazide (Mancia et al., 2023). The desired drug names were searched using MeSH terms to ensure completeness. The PT associated with OA were retained and analysed in subgroups based on population-specific, gender, age, and drug use patterns. The time to onset of drug-related OA finding signals was also calculated. Time to onset was defined as the time interval between the date of AE onset and the date of initiation of medication use. The Weibull shape parameter (WSP) test was used to analyse the onset time (Abe et al., 2016). The time to onset data was evaluated based on the median, quartiles, and WSP test. The incidence of AEs after treatment initiation depends on the drug’s mechanism of action and usually varies over time. In contrast, the incidence of AEs unrelated to drug therapy remains constant. Early failure type curves are characterised by β values less than 1 and a 95% CI also less than 1, indicating a decreasing hazard over time. Random failure type curves, on the other hand, have β values equal to or close to 1 and a 95% CI containing 1, indicating a steady and consistent hazard over time. Finally, wear-off type curves have β values greater than 1 and a 95% CI not containing 1, indicating an increasing hazard (Sauzet et al., 2013; Nakamura et al., 2015). A Logistic model was employed to substantiate the hypothesis that age and gender are risk factors for the occurrence of AEs.

### 2.3 Statistical analysis

Pharmacovigilance signals were measured using proportional disproportionality analysis. Adverse event signals were identified and defined using the reported odds ratio (ROR), which was considered to have occurred if a drug-induced adverse event was recorded as greater than or equal to 3 and the lower limit of the ROR confidence interval was greater than 1 (Sakaeda et al., 2013; Tian et al., 2022). The ROR and its 95% confidence interval were calculated using the formula below

$$\text{ROR}=\frac{\left(a/c\right)}{\left(b/d\right)}$$

$$95\%\text{CI}=e^{\ln\left(\text{ROR}\right)\;}\pm 1.96\sqrt{\left(\frac{1}{a}+\frac{1}{b}+\frac{1}{c}+\frac{1}{d}\right).}$$

**FIGURE 1:**
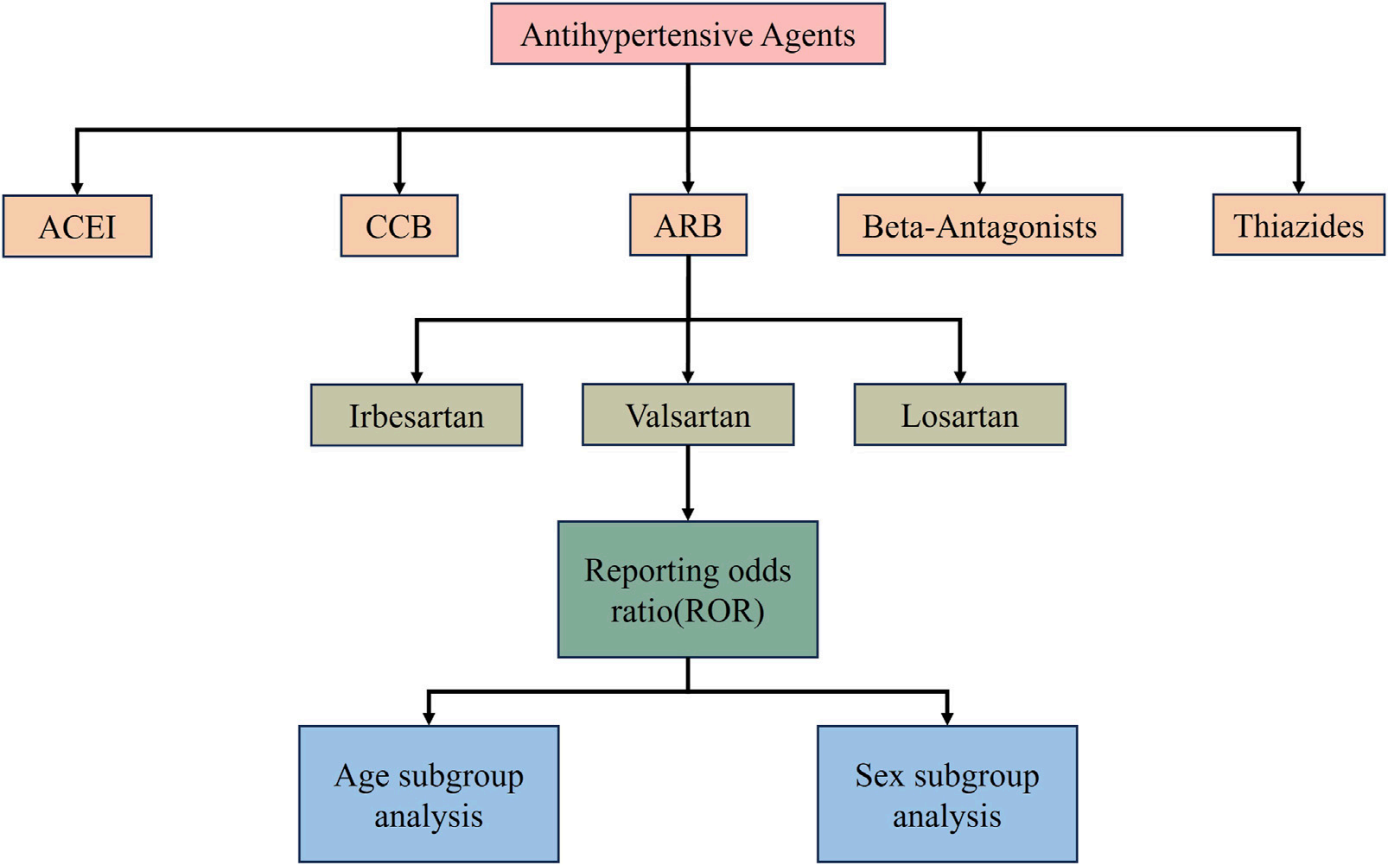
Research retrieval analysis flowchart.

The higher the ROR value, the stronger the adverse effect signal, indicating a stronger correlation between the drug and the AE. Baseline data were described by frequency counts and frequencies (Jain et al., 2023; Sharma et al., 2023). Data were collated and statistically analysed using R (version 4.3.2) and the accompanying Rtools and Rstudio versions (Figure 1).

## 3 Results

### 3.1 Five classes of antihypertensive drugs correlate with the occurrence of OA AEs

**FIGURE 2:**
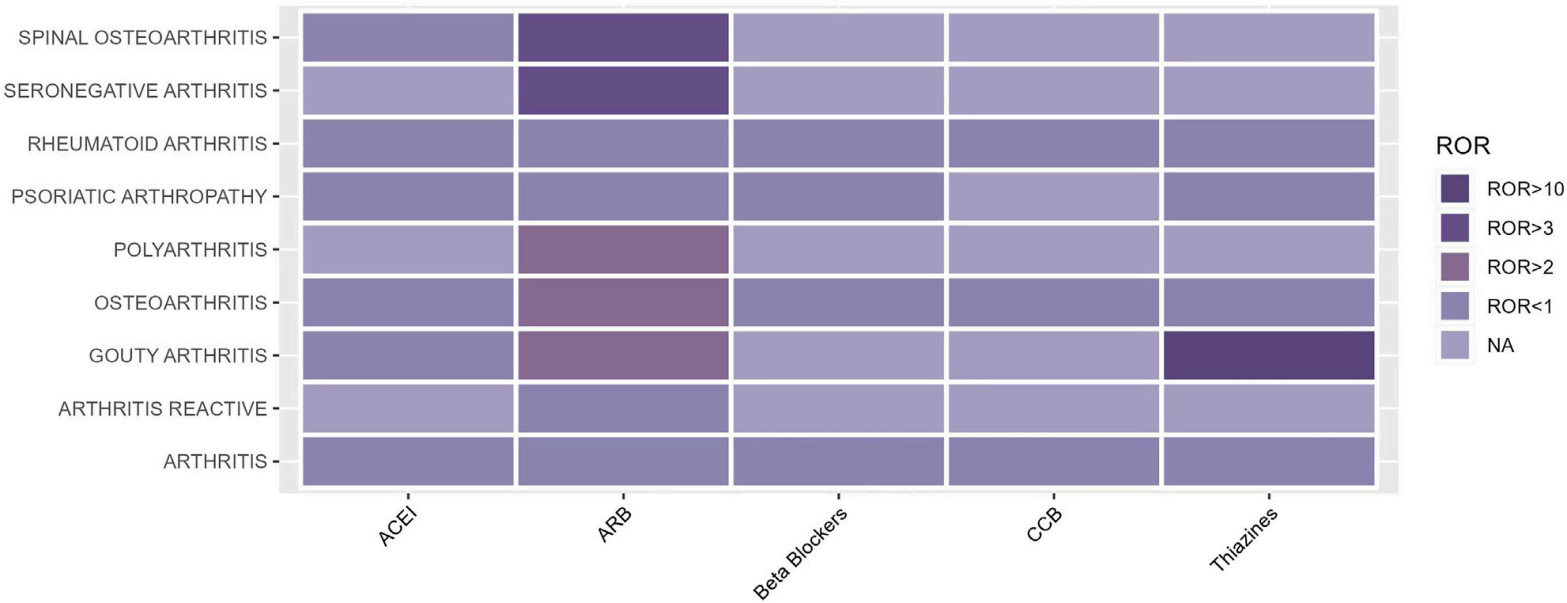
Statistical chart of OA AEs signals associated with each type of antihypertensive drug.

The occurrence of OA AEs in patients treated for hypertension was investigated in the FAERS database from 2004 to 2023 using the descriptive methodology described above. OA AEs in PT included spinal osteoarthritis, seronegative arthritis, rheumatoid arthritis, psoriatic arthropathy, polyarthritis, osteoarthritis, gouty arthritis, arthritis, and reactive arthritis. Figure 1 displays the results. The study found that both ARBs and thiazides have adverse effects related to arthritis. Positive AEs were observed for several ARB drugs, including spinal osteoarthritis (ROR 3.24, 95%CI 2.56–4.11), seronegative arthritis (ROR 3.96, 95%CI 1.88–8.35), polyarthritis (ROR 1.71, 95%CI 1.09–2.68), and osteoarthritis (ROR 2.27, 95%CI 1.99–2.59). Thiazide-related AEs were positively associated with gouty arthritis (ROR 24.68, 95%CI 13.95–43.68). As hyperuricaemia and gout are well-documented adverse effects of hydrochlorothiazide drugs (Hueskes et al., 2012; Dhayat et al., 2023), this study will focus on the OA AEs from ARBs (Table 1; Figure 2).

**TABLE 1:** Antihypertensive drugs-related OA AEs.

Drugs	AEs	a value	ROR	95%CI
Thiazine drugs	Gouty arthritis	12	24.68	13.95–43.68
ARB drugs	Osteoarthritis	219	2.27	1.99–2.59
	Spinal osteoarthritis	69	3.24	2.56–4.11
	Polyarthritis	19	1.71	1.09–2.68
	Seronegative arthritis	7	3.96	1.88–8.35

### 3.2 Statistical baseline data on valsartan and OA AEs

**FIGURE 3:**
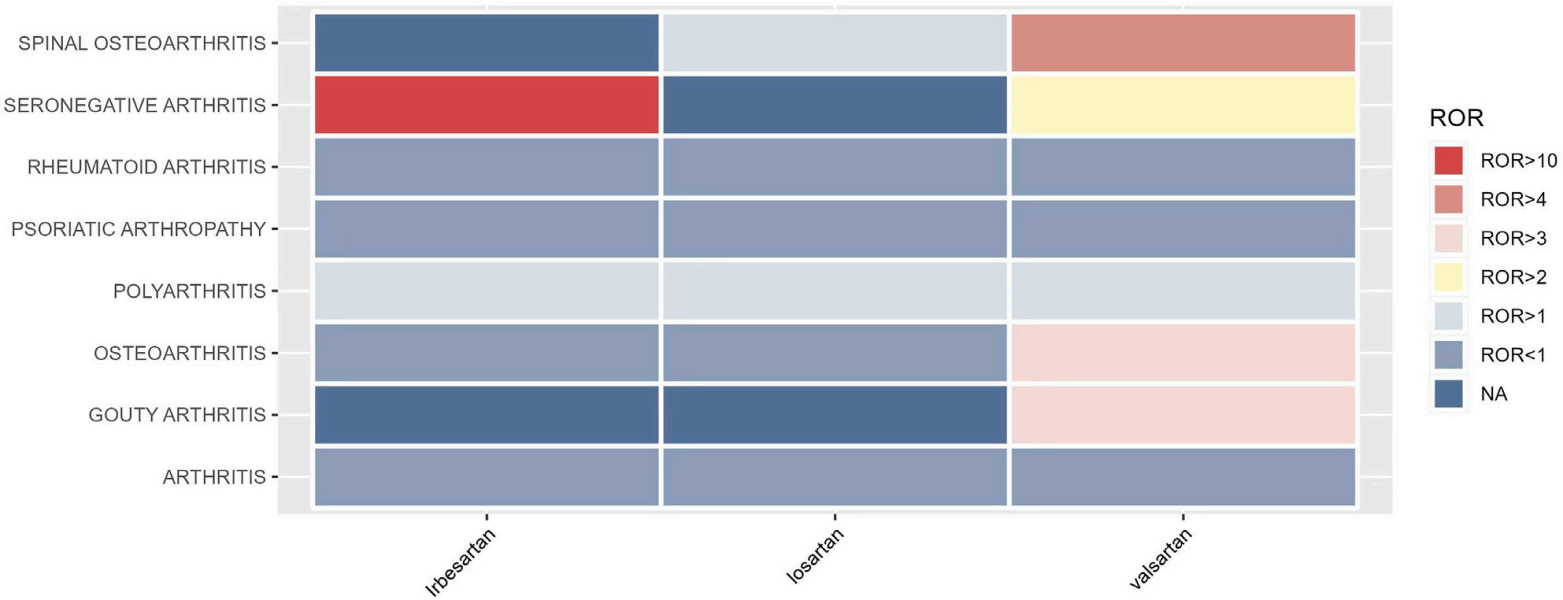
Signal intensity of OA AEs associated with three ARB drugs.

**FIGURE 4:**
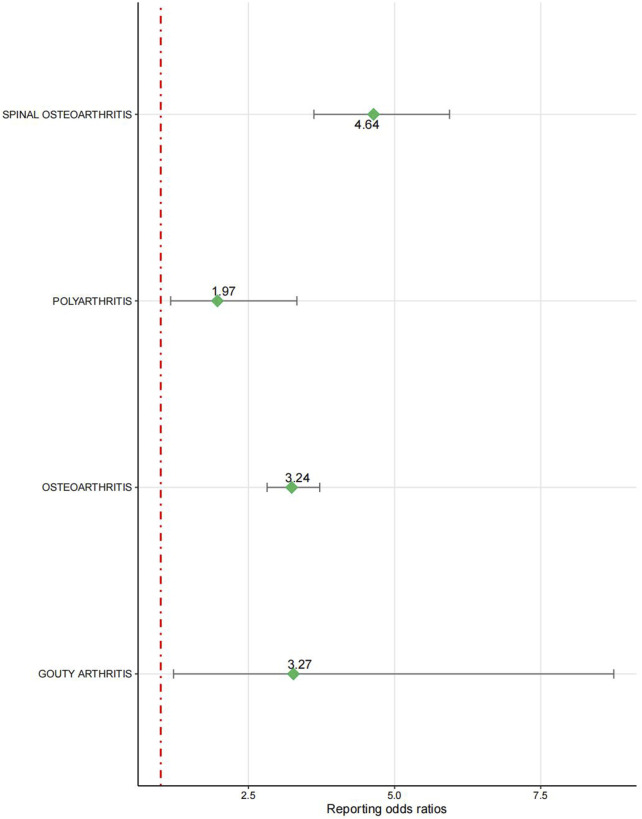
Forest plot of valsartan-related OA AEs.

The signal intensity of OA AEs was counted for each of the three drug classes (Table 2; Figure 3). Irbesartan showed a positive signal for seronegative arthritis (ROR 16.53, 95%CI 6.18–44.18), while valsartan showed a positive signal for osteoarthritis (ROR 3.24, 95%CI 2.82–3.72), spinal osteoarthritis (ROR 4.64, 95%CI 3.62–5.94), polyarthritis (ROR 1.97, 95%CI 1.17–3.33), and gouty arthritis (ROR 3.27, 95%CI 1.22–8.75) (Figure 4). In contrast, no arthritis-associated signals were found for cloxartan.

**TABLE 2:** ARBs-related OA AEs.

Drugs	AEs	a value	ROR	95%CI
Valsartan	Osteoarthritis	199	3.24	2.82–3.72
	Spinal osteoarthritis	63	4.64	3.62–5.94
	Polyarthritis	14	1.97	1.17–3.33
	Gouty arthritis	4	3.27	1.22–8.75
Irbesartan	Seronegative arthritis	4	16.53	6.18–44.18

### 3.3 General characteristics of adverse event reports associated with OA occurring at valsartan

After observing that valsartan produced the highest number of OA-related positive PT, we analysed the valsartan drug data for age and subgroups. We began by counting the valsartan drug data in general (Table 3). A total of 22,288 reports were recorded from the first quarter of 2004 to the third quarter of 2023. It was found that valsartan-related OA AEs were more prevalent in women than in men. Of the gender-specific reports, 12,026 were from women and 8,308 were from men, resulting in a ratio of 1.45 women to 1 man. The highest number of reports came from individuals weighing between 50–100 kg, accounting for 20% of the total number of reports. The age group with the highest number of reports was 65–85 years old, representing 55.6% of the known age reports, which is consistent with the prevalence of hypertension combined with OA. The reporting population consisted mainly of consumers and physicians, who submitted 8,153 reports (43.2%) and 4244 reports (22.5%), respectively. The reporting population consisted mainly of consumers and physicians, who submitted 8,153 reports (43.2%) and 4244 reports (22.5%), respectively. The reporting population consisted mainly of consumers and physicians, who submitted 8,153 reports (43.2%) and 4244 reports (22.5%), respectively. These two groups accounted for more than half of the reports (Table 3).

**TABLE 3:** Baseline data reported for valsartan-related OA AEs.

Characteristic	Case(N)	Proportion (%)
Sex		
Female	12,026	54.00
Male	8,308	37.30
Unknown	1954	8.80
Weight (kg)		
<50	436	2.00
50–100	4,449	20.00
>100	717	3.20
Unknown	16,686	74.90
Age (year)		
<18	83	0.40
18–64.9	3,803	17.10
65–85	6,077	27.30
≥85	977	4.40
Unknown	11,348	50.90
Reported person		
Non-healthcare professional Consumer (CN)	11,494	51.60
Health professional (HP)	700	3.10
Lawyer (LW)	519	2.30
Health professional Physician (MD)	5,089	22.80
Other health professional (OT)	2054	9.20
Pharmacist (PH)	1,310	5.90
Unknown	1,122	5.00

### 3.4 Age, gender and temporal characteristics of valsartan-related OA AEs

**FIGURE 5:**
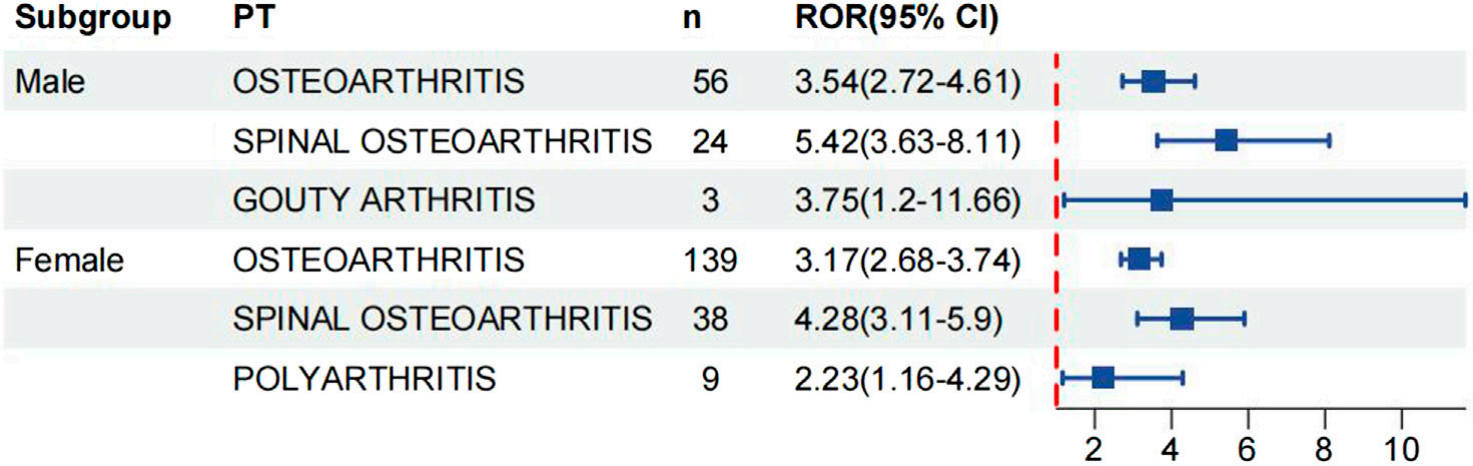
Gender subgroup analysis of reports of valsartan-associated OA AEs.

**FIGURE 6:**
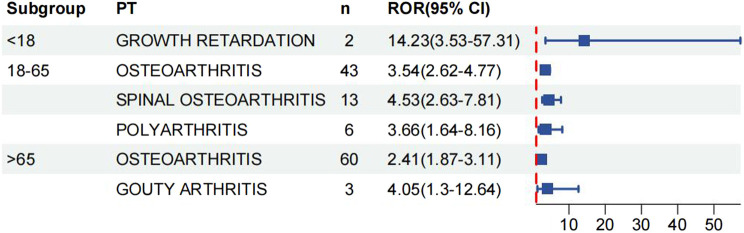
Age subgroup analysis of reports of valsartan-associated OA AEs.

We analysed age and gender subgroups for adverse reactions associated with valsartan and found that the highest number of OA cases was reported in male patients (n = 56, ROR 3.54, 95% CI 2.72–4.61). The most commonly reported condition in females was OA, with a higher incidence than in males (n = 139, ROR 3.17, 95%CI 2.68–3.74) (Figure 5). In the age subgroup analysis for individuals under 18 years old, the only skeletal muscle-related adverse event was growth retardation, with a total of two reports (n = 2, ROR 14.23, 95%CI 3.53–57.31). The report shows that OA was the most commonly reported condition in both age groups, with 43 reports and a ROR of 3.54 (95%CI 2.62–4.77) in the 18–65 year olds and 60 reports and a ROR of 2.41 (95%CI 1.87–3.11) in the over-65 year olds (Figure 6) (23, 95%CI 3.53–57.31). Logistic regression modelling was used to investigate the impact of age and gender on the occurrence of OA AEs. The results showed that gender had a significant effect on the occurrence of valsartan-related OA AEs, with males being a protective factor. However, no significant effect of age on the occurrence of AEs was observed (Table 4). Finally, we analysed the trend of each post-traumatic stressor over time using the Weibull distribution. We found that polyarthritis was part of the wear-and-tear inefficacy model, indicating that the risk of polyarthritis associated with valsartan increased with time. In contrast, all other forms of arthritis and the overall trend belonged to the follow-through failure type (Table 5).

**TABLE 4:** Logistic regression analysis of gender subgroups reporting valsartan-associated OA AEs.

	Arthritis	Non-arthritis	OR (univariable)	OR (multivariable)
Female	6,128 (98.9)	71 (1.1)	Reference	Reference
Male	4,680 (99.5)	25 (0.5)	0.46 (0.29–0.72, *p* = 0.001)	0.45 (0.28–0.71, *p* = 0.001)

**TABLE 5:** Weber analysis of reports of valsartan-associated OA AEs.

AEs	Case reports	Scale parameter: α(95% CI)	Shape parameter: β(95% CI)	Type
Osteoarthritis	13	531.94 (172.64–891.25)	0.85 (0.49–1.22)	Random failure type
Spinal Osteoarthritis	8	598.51 (103.11–1093.91)	0.89 (0.40–1.37)	Random failure type
Polyarthritis	3	944.10 (825.39–1062.81)	9.45 (0.35–18.54)	Wear type fault
Total	25	624.92 (357.46–892.38)	0.97 (0.66–1.27)	Random failure type

## 4 Discussion

The prevalence of hypertension in patients with OA is on the rise. Studies have demonstrated that hypertension exacerbates the progression of OA and heightens the likelihood of adverse outcomes (Hall et al., 2016; Zhang et al., 2017; Veronese et al., 2018). Therefore, it is crucial to identify the risks and adverse effects of hypertensive drugs on OA. Administering appropriate hypertensive drugs to control blood pressure in patients with OA can enhance their quality of life and survival rate. Antihypertensive drugs have been associated with the progression or symptoms of OA. However, no study has examined the relationship between antihypertensive drugs and OA in terms of adverse drug reactions. This study searched and analysed five classes of representative first-line drugs for the treatment of hypertension through the FAERS database. The study found an association between ARBs and thiazides with OA, while CCBs, thiazide diuretics, and β-blockers were not associated with OA. Further investigation revealed that the positive signals were mainly from valsartan drugs. Stratification by age and gender showed that the positive signals were stronger in women and people over 65 years of age, while men were less likely to experience ARB-associated OA AEs. We found that the occurrence of polyarthritis associated with valsartan increased with the duration of drug administration, according to Weibull distribution analysis.

Articular cartilage consists of chondrocytes and an extracellular matrix comprising type II collagen and proteoglycans (Cheng et al., 2013). It is a non-vascular tissue, which limits its self-repairing ability after injury, thereby increasing the risk of OA (Alford and Cole, 2005; Liu et al., 2017; Powers et al., 2021). Therefore, blood vessels play a crucial role in cartilage repair. As OA is a chronic disease, it is prevalent among the elderly population and is often associated with hypertension and other diseases (Eymard et al., 2015; Schlenk et al., 2021). Hypertension can cause vascular damage, which affects the inflammatory response and oxidative stress in the body. This, in turn, affects the blood supply and nutrition of skeletal tissues (Fernandes and Valdes, 2015; Mosquera et al., 2019; Merkely et al., 2021). It is important to control hypertension for the quality of life and prognosis of patients with OA (Smith and Cooper-DeHoff, 2019; Zhang et al., 2023). A number of studies have demonstrated that antihypertensive drugs can influence bone metabolism (Song et al., 2024a; Song et al., 2024b; Ma et al., 2024), with a complex relationship also emerging with OA (Bae et al., 2015; Uzieliene et al., 2019). Therefore, it is crucial to determine whether the drugs have adverse effects related to OA. This study covers six classes of first-line drugs used to treat hypertension. The study found that CCBs, ACEIs, and β-blockers do not have OA-associated adverse effects, while ARBs and thiazide diuretics have high signals.

Valsartan is a member of the ARB drugs. It acts by blocking the angiotensin II receptors, thereby dilating the blood vessels and lowering blood pressure. Angiotensin II, the key molecule of RAS, acts *in vivo* by binding to angiotensin II type 1 (AT1) and type 2 (AT2) receptors (Peach, 1977). It is therefore possible that ARB drugs play a role in the regulation of OA by modulating the level of synovial inflammation and the release of synovial inflammatory factors (Terenzi et al., 2017). Recent studies have demonstrated that angiotensin II is expressed in synovial tissues of both humans and animals and is involved in the pathogenesis of OA (Liu et al., 2016; Forrester et al., 2018; Wang et al., 2018). It has been demonstrated that ARB can facilitate the expression of transforming growth factor-β (TGF-β) (Wu et al., 2016; Zou et al., 2022). Although TGF-β is essential for articular cartilage homeostasis (Blaney Davidson et al., 2007), it has also been found that an excessive amount of TGF-β can be produced during OA (Bakker et al., 2001; van de Laar et al., 2011). In addition, intra-articular knee injections of TGF-β1 induced OA in rats (Serra et al., 1997; Itayem et al., 1999; Shen et al., 2013). Furthermore, it has been demonstrated that ARB drugs may facilitate chondrocyte hypertrophic senescence during skeletal development in mice (Chen et al., 2015). Some studies have further explored the mechanism and found that ARB can participate in OA regulation through the activation of NF-κB and phosphorylation of JNK (Sampson et al., 2016; Yu et al., 2016; Yamagishi et al., 2018). The NF-κB signalling pathway plays a pivotal role in the release of inflammatory factors, which is a crucial step in the progression of OA (Saito and Tanaka, 2017; Cao et al., 2021). Additionally, the phosphorylation of JNK has been observed to result in the enhanced secretion of matrix metalloproteinase 13 (MMP13), a protein that accelerates the breakdown of cartilage tissue (Lin et al., 2021; Sun et al., 2023). Furthermore, evidence indicates that ARB drugs can influence the progression of OA by activating vascular endothelial growth factor (VEGF) molecules (Chen et al., 2017; MacDonald et al., 2018). Our study found that valsartan had a positive signal for OA AEs. It is therefore recommended that valsartan be used with caution when patients with OA and hypertension require treatment with ARBs. However, higher quality RCTs and mechanism studies are needed to provide stronger evidence.

Age and gender are widely acknowledged as risk factors for OA (Felson et al., 2000; Wluka et al., 2000). Age is one of the strongest risk factors for OA in the elderly due to biological changes such as loss of muscle strength, cartilage wear and tear, and deterioration of proprioception that occur with age (Felson and Zhang, 1998; Helmick et al., 2008). In this study, it was also found that the number and proportion of positive reports were highest among people above 65 years of age, compared to other age groups. Age may exacerbate the manifestation and probability of valsartan-related OA AEs. Additionally, women are at a higher risk of developing OA and experiencing more severe symptoms than men (Hannan et al., 1990; Srikanth et al., 2005). This may be due to the effect of endogenous oestrogens and changes during menopause in women. In addition, our findings suggest that females may be more susceptible to valsartan-related OA AEs. However, the data on hypertension revealed a higher number of records for female patients than for male patients. Further high-grade studies are required in the future to investigate the impact of gender on valsartan-related OA AEs. A study demonstrated that the prevalence of hypertension was lower in premenopausal women than in men of the same age, and increased significantly in postmenopausal women (Ji et al., 2020). Furthermore, the prevalence of hypertension is higher in women than in men over the age of 65 years (O'Keeffe et al., 2018). We concluded from Weibull’s analysis that the incidence of AEs associated with valsartan in OA patients increased with the duration of administration. This reinforces our recommendation that valsartan should be used with caution in patients with OA.

Apart from the above, hydrochlorothiazide and irbesartan also demonstrated positive effects. Hydrochlorothiazide, in particular, significantly increases the risk of gout by affecting uric acid excretion (Schrijver and Weinberger, 1979; Dhayat et al., 2023). As a result, this study also found a positive association between hydrochlorothiazide and gouty arthritis. The present study did not investigate or discuss in detail the relationship between irbesartan and OA due to the lack of relevant studies and the small number of reports associating irbesartan only with seronegative arthritis. Therefore, further clinical and experimental studies are needed to establish the relationship between irbesartan and OA. Furthermore, different conclusions have been reached in studies on the relationship between CCBs and β-blockers and OA. For instance, some studies have proposed that CCBs can decelerate the progression of OA by antagonising intra-articular calcium channels (Takamatsu et al., 2014; Bertram et al., 2016). However, other studies have suggested that CCBs may worsen pain and joint stenosis in OA patients (Daniilidis et al., 2015; Li M. et al., 2021). Additionally, there are conflicting findings regarding the effects of β-blockers on OA (Martin et al., 2015; Valdes et al., 2017). The results of this study indicate that there are no discernible positive signals for OA AEs for any of the aforementioned pharmaceutical agents. Further high-quality clinical and mechanistic studies are needed to establish the relationship between them.

Furthermore, initial guidelines recommended monotherapy for the control of hypertension. However, an increasing number of studies have shown that blood pressure is a multi-regulatory variable involving multiple pathophysiological processesand that patient compliance with monotherapy is poor (Berra et al., 2016; Rea et al., 2018). Therefore, monotherapy may not be sufficient to control blood pressure in most patients (Bakris et al., 2014). Among them, ACEI or ARB in combination with CCB or thiazide is the most recommended combination regimen in the guidelines (Mancia et al., 2023). It should be noted that ARBs, β-blockers and thiazide diuretics are all associated with hyperuricemia (Choi et al., 2012; Bardin and Richette, 2017). Therefore, these drugs should be avoided when treating patients with gouty arthritis and hypertension. In addition, the combination of ARBs and ACEIs or two ARB drugs increases the likelihood of nephrotoxicity and acute renal failure, which is also noteworthy (Ontarget et al., 2008; Parving et al., 2009). However, there is a lack of clinical trials on the use of combinations in the treatment of patients with arthritis. The amount of data on co-medication in the FAERS database is also limited, making it difficult to investigate whether co-medication causes OA AEs. In future studies, we will focus on the effect of ARB combination therapy on the occurrence of OA AEs.

There are limitations to this study. Firstly, we only included five first-line and representative anti-hypertensive drugs, which may introduce bias in the results. Secondly, the accuracy of statistical results may be biased during the initial data entry stage due to the self-reporting nature of the FAERS database. Inadequate case reporting can also significantly impact study results. Furthermore, the majority of cases recorded in the FAERS system are from Europe and the United States, indicating a need for more global data entry.

## 5 Conclusion

Through comprehensive and systematic analysis of the FAERS data, we have identified a strong association between five classes of first-line antihypertensive drugs and OA-related side effects. We have also assessed the severity of AEs across different populations, age groups, and genders. Specifically, we have found a strong adverse effect signal for the valsartan drug in the ARB class. Subgroup analyses showed that the population over 65 years and females had the highest number of positive reports. Females appear to be more susceptible to valsartan-related OA AEs, but higher quality studies are needed to confirm this. Additionally, the incidence of valsartan-associated OA AEs increased with increasing duration of dosing. Therefore, We recommend that patients with OA combined with hypertension should use ARBs with caution, especially valsartan.

## Data Availability

The raw data supporting the conclusions of this article will be made available by the authors, without undue reservation.

## Abbreviations

OA, Osteoarthritis; ARB, Angiotensin receptor blocker; ACEI, Angiotensin converting enzyme inhibitors; AT, Angiotensin; CCB, Calcium channel blockers; ARNI, Angiotensin receptor-neprilysin inhibitor; TGF-β, Transforming growthfactor β; RCT, Randomized controlled trial.
